# Ex Vivo Comparison of the Diagnostic Performance of Two-Dimensional and Three-Dimensional Three-Tesla Magnetic Resonance Imaging Sequences in Depicting Normal Articular Cartilage in Equine Stifle Cadavers

**DOI:** 10.3390/ani14010015

**Published:** 2023-12-19

**Authors:** Annika Seidler, Anton Aßmann, Paul R. Torgerson, José Suárez Sánchez-Andrade, Andrea Bischofberger

**Affiliations:** 1 Equine Hospital, Vetsuisse-Faculty, University of Zürich, 8057 Zürich, Switzerland; 2 Section of Veterinary Epidemiology, Vetsuisse-Faculty, University of Zürich, 8057 Zürich, Switzerland; 3 Equine Hospital, Clinic of Diagnostic Imaging Vetsuisse-Faculty, University of Zürich, 8057 Zürich, Switzerland

**Keywords:** horse, stifle joint, MRI, 2D sequences, 3D sequences, articular cartilage

## Abstract

**Simple Summary:**

At present, it is difficult to evaluate the cartilage of an equine stifle with the most accessible imaging modalities, such as ultrasonography and radiography. Magnetic resonance imaging (MRI) has been established as the optimal noninvasive modality for a global evaluation of joints in humans. With technical advances, MRI is becoming more available for equine patients as well. Currently there is no general consensus on the optimal sequence for cartilage evaluation even though there are many possibilities. This study compares a routinely used two-dimensional (2D) sequence to four different three dimensional sequences (3D). Measuring the cartilage thickness in 30 different regions of interest (ROI) in each sequence and comparing them to macroscopic measurements showed that the 3D sequences were more accurate than the 2D sequence. Specifically, the 3D VIEW T2W HR and T1W VISTA SPAIR depicted cartilage thickness most accurately. Knowing the accuracy of different sequences improves the evaluation of equine cartilage and the early detection of cartilage pathologies. This promotes MRI as a noninvasive imaging modality for horses suffering from stifle lameness with no findings using conventional imaging methods. Furthermore, since 3D sequences are more accurate in depicting cartilage, they may replace 2D sequences, shortening scanning time.

**Abstract:**

The objective of this study was to compare articular cartilage thickness observed in the different 2D and 3D sequences to the cartilage thickness of the equine stifle in cadavers to determine the accuracy of each sequence. The study was conducted as a blinded laboratory study using seven equine stifle specimens. The 2D (T2W TSE) and 3D (3D VIEW T2W HR, T2 3D mFFE, T1W VISTA SPAIR, 3D PDW SPAIR) 3-tesla MRI sequences of each stifle were obtained. Cartilage thickness was measured at 30 locations on MRI and on gross pathology. Thickness measurements were compared using a Bland–Altman plot and post hoc analysis tests. The 3D sequences were found to be generally more accurate than the 2D sequence (*p* < 0.001). The smallest difference to macroscopic measurements was observed in the 3D VIEW T2W HR and T1W VISTA SPAIR sequences with no statistical difference between each other. Knowing the accuracy of different sequences will improve the evaluation of equine cartilage and the early detection of cartilage pathologies. This would promote MRI as a noninvasive imaging modality for horses suffering from stifle lameness with no findings in conventional imaging methods. Furthermore, since 3D sequences seem to have better accuracy in depicting cartilage, they may replace 2D sequences, thereby shortening scanning times.

## 1. Introduction

Most stifle pathologies can be classified as having developmental or traumatic etiologies and can cause significant lameness [1]. In many cases, osteoarthritis may develop as a secondary problem, but it can also occur primarily without any underlying pathology being identified. A distinctive feature of osteoarthritis is articular cartilage damage and degeneration [1].

The accurate visualization of articular cartilage is a challenge. Conventional imaging modalities such as radiography and ultrasonography have limitations. The former does not directly depict cartilage and the latter has user-dependent accuracy, often being incongruent with the macroscopic pathological changes and is not able to identify the weight-bearing articular surface [2,3]. Diagnostic arthroscopy has high sensitivity to the cartilage surface that can be visualized, but a complete exploration of the joint is not achievable due to the unfeasibility of joint distraction [1,2].

Newer technology such as computed tomography (CT) is capable of the significantly better detection of the focal bone production than radiography, and thus provides a better indication of osteoarthritis and probable cartilage damage [2]. To visualize cartilage, CT arthrography (CTA) using a contrast medium is necessary [2]. In humans, CTA has been indicated as the gold standard imaging method for the morphological evaluation of the cartilage surface [2]. In equine carpal joints, CTA has been shown to have the highest sensitivity for artificial articular cartilage defects when compared to plain CT, plain magnetic resonance imaging (MRI), and MRI arthrography [4]. The equine stifle has been described, however, as being more difficult to evaluate using CTA due to the firm apposition of the cartilage of the femoral and tibial condyles to the menisci [5].

In humans, MRI has been established as the optimal non-invasive diagnostic method for evaluating articular cartilage [6,7]. With advancing technology, stifle MRI is becoming more readily available in equine clinics, although it still remains a challenge [8]. Although there are many possibilities, currently no ideal sequence for evaluating cartilage has been determined [2].

Two-dimensional (2D) T2-weighted and proton density-weighted (PDw) spin-echo (SE) and fast spin-echo (FSE) MRI sequences have been used routinely for morphological evaluation because of the high in plane spatial resolution, tissue contrast resolution and the possibility of performing the morphological evaluation of other articular structures such as menisci, ligaments, and subchondral bone [2,7,9,10]. Generally, fat suppression can be added to sequences and has been shown to improve contrast at the cartilage-to-bone interface [7]. PD-weighted images have a higher internal cartilage signal than T2-weighted images. However, they are susceptible to magic angle effects. To improve tissue contrast resolution between synovial fluid, articular cartilage, and subchondral bone interface, while also reducing susceptibility to the magic angle effects, intermediate-weighted sequences can be used [7]. The drawbacks of 2D imaging are the anisotropic voxels, section gaps, partial volume effects, and longer acquisition times [2,7,9,10].

In addition to two-dimensional techniques, three-dimensional (3D) MRI sequences are also available. Gradient-recalled echo (GRE), as a 3D imaging sequence, has been shown to improve the detection of cartilage abnormalities [2,9]. However, scanning times are long and contrast tissue resolution is lower than in spin-echo imaging [2,9]. Recently, 3D intermediate-weighted fast and turbo spin-echo (FSE and TSE) sequences have been shown to have high agreement with 2D techniques in humans, even for other previously mentioned articular structures [9,11]. A study comparing 3D-FSE to 3D-GRE imaging concluded that FSE had a good diagnostic performance for cartilage lesions, comparable to GRE sequences [12]. Three-dimensional MRI sequences permit multiplanar reconstruction using isotropic voxel dimensions. This shortens the scanning time since only one plane needs to be acquired [2,9,11]. Slices in 3D sequences are very thin compared to 2D, allowing for smaller articular cartilage lesions to be detected [2]. A drawback of 3D reconstruction may be a decreased signal-to-noise ratio and a lower articular cartilage signal resulting in lower image quality [2].

In human studies, 3D MRI techniques show promise for replacing conventional 2D sequences [9,11,12]. In horses, studies on stifle MRI are limited, and no studies have been performed comparing 3D FSE and 2D TSE sequences. The objective of this study is to determine the diagnostic performance of 2D (T2W TSE) and 3D MRI (3D VIEW T2W HR, T2 3D mFFE, T1W VISTA SPAIR, 3D PDW SPAIR,) sequences for measuring articular cartilage thickness in the equine stifle compared to gross pathology. It was hypothesized that the diagnostic performance of 3D MRI sequences would be at least as accurate as conventional 2D MRI sequences in depicting articular cartilage with the advantage of decreasing overall scanning times.

## 2. Materials and Methods

### 2.1. Specimen Collection

After obtaining the owners’ consent, the stifle joints (*n* = 7) of adult warmblood horses euthanized for non-orthopedic reasons were collected within 24 h of euthanasia and then stored at −28 °C. All limbs were thawed over 24 h to room temperature, then all limbs underwent standard femoropatellar and femorotibial joint arthroscopy to evaluate the articular surface prior to MRI. Only stifle joints with no signs of joint disease were further included in the study.

### 2.2. Imaging Procedure

An MRI examination was performed using a 3-tesla MRI scanner (Philips Ingenia, Philips AG, Zurich, Switzerland). The obtained MRI sequences, planes, and acquisition parameters are shown in Table 1. Limbs were positioned in lateral recumbency in slight flexion with the toe facing away from the gantry and scanned using a dStream Torso coil (Philips AG, Zurich, Switzerland). To acquire the 2D images, the sagittal plane was oriented along the trochlear ridges, the dorsal along the condyles, and the transvers according to the long axis of the femur.

### 2.3. Image Analysis

Images were reviewed by two of the authors (J.S.S. and A.S.), and a consensus was reached using a diagnostic workstation and professional medical imaging software (Horos, v2.0.0 RC5). The reviewers received a random order in which the images had to be reviewed. Reviewers were allowed to alter the window level and width, zoom, and evaluate any of the sequences within the individual study, but not compare the individual studies.

#### 2.3.1. Femoropatellar Joint

**Figure 1:**
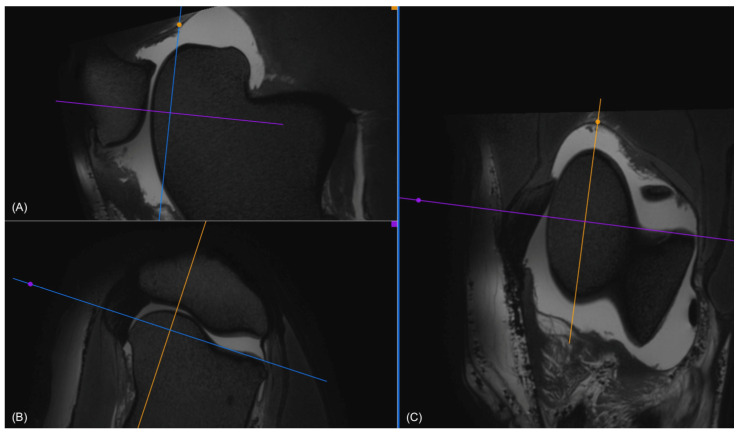
(**A**) Sagittal, (**B**) transverse, and (**C**) dorsal view in 3D VIEW T2-weighted images of a stifle, where the regions of interest (ROIs) were determined for the medial trochlear ridge. In the transverse view (**B**), the dorsal plane (blue) was oriented to be parallel to the joint space. In the dorsal view (**C**), the sagittal plane (yellow) was oriented according to the long axis of the medial ridge. This was repeated for lateral and axial measurements by orienting the sagittal plane (yellow), according to the long axis of the lateral ridge and the trochlear groove, respectively.

**Figure 2:**
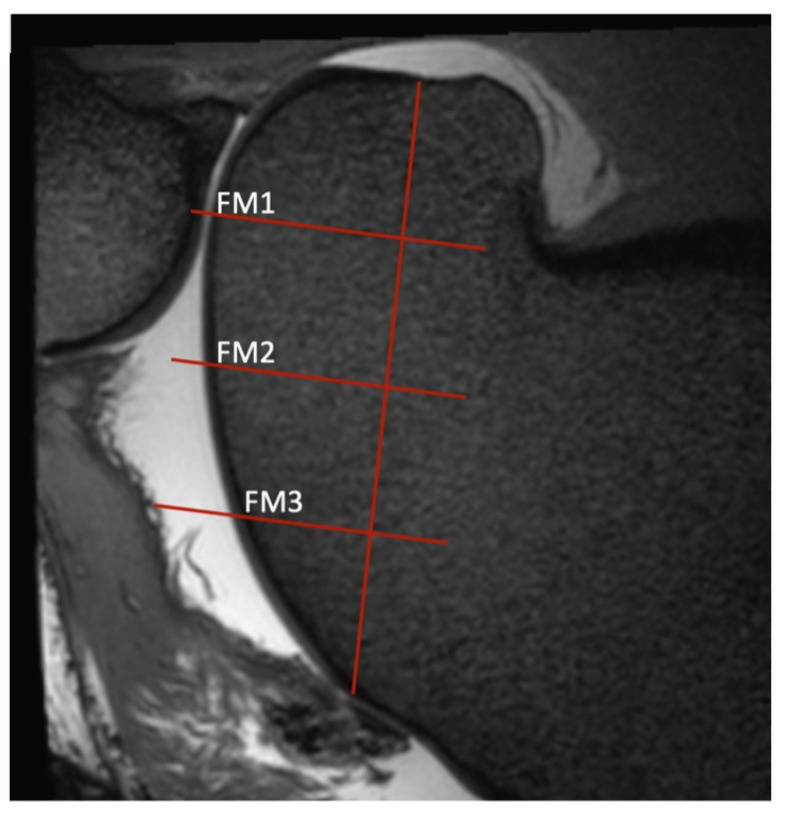
Sagittal view of a T2-weighted image of the medial trochlear ridge of a stifle, as determined by the procedure described in Figure 1. Three regions of interest (ROIs) were defined. In the sagittal view, a line was drawn from the notch where the trochlea ends and the condyle starts up to where the trochlear cartilage ends. This stretch was then quartered, resulting in three lines perpendicular to the original line. Where these lines intersect with the articular cartilage, ROIs (FM1, FM2, and FM3) were determined and the cartilage thickness was measured. The same process was repeated for the lateral dorsal view in the respective sagittal planes. For the lateral measurements, the middle of the origin of the lateral digital extensor tendon was used as the starting point of the vertical line and the proximal edge of the cartilage as the end. For the trochlear groove, the distal and proximal cartilage edges were used as starting and end points. As on the medial trochlear ridge, these two lines through the lateral trochlear ridge and the central groove were quartered with lines perpendicular to the first, resulting in three ROIs each.

**Figure 3:**
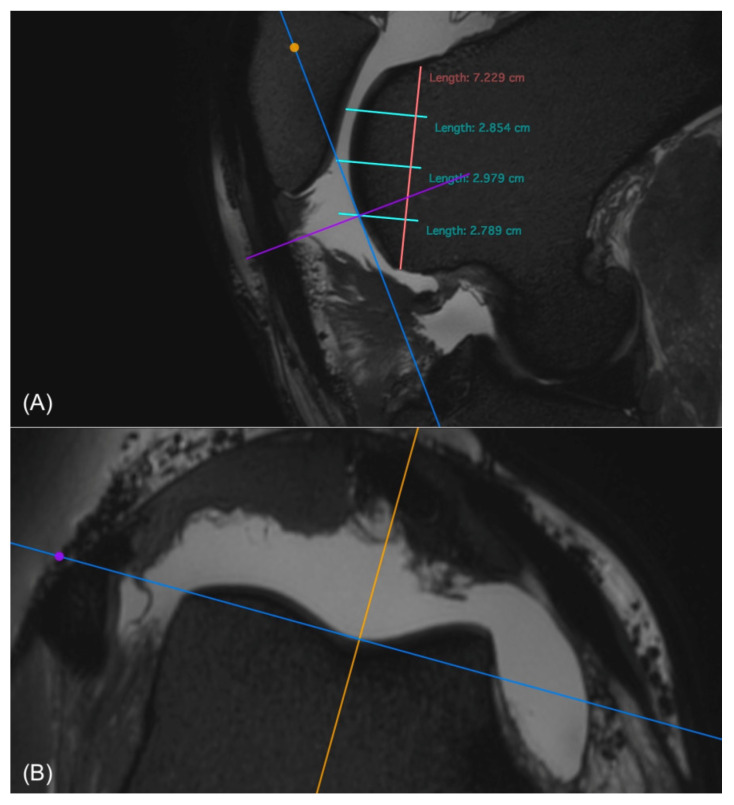
(**A**) Sagittal and (**B**) transverse view of the trochlear groove in a 3D T2-weighted image where the regions of interest (ROIs) were determined. The ROIs are marked as described in Figure 1 and Figure 2 (**A**). To measure the cartilage thickness in the transverse image (**B**) at the same ROI, the cross of the transverse (purple) and dorsal (blue) planes was placed at the ROI in the sagittal view (**A**). This automatically places the plane cross on the same ROI in the transverse view (**B**). Care was taken to place the transverse plane (purple) perpendicularly to the cartilage surface at the point of measurement. This was carried out for all ROIs in the femoropatellar joint. For the femorotibial joint the dorsal view was used instead of the transverse.

**Figure 4:**
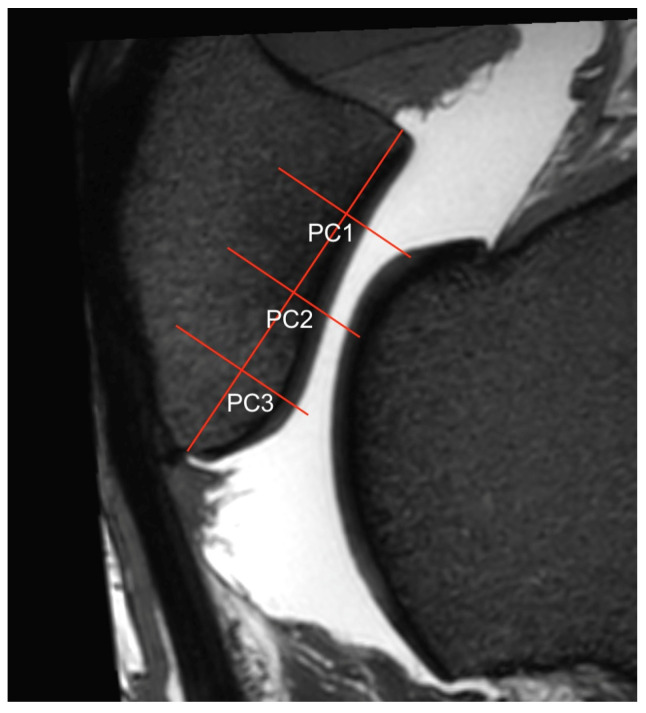
Sagittal view of the central ridge of the patella in a 3D T2w VIEW sequence where the regions of interest (ROIs) PC1, PC2, PC3) were measured. To determine the ROIs of the patellar cartilage, the same sagittal orientations were used as for the trochlea (Figure 1). In the transverse view, the dorsal plane was adjusted to be parallel to the patellar joint surface at the specific ROIs. This was readjusted for the ridge and each groove of the patella to account for the different orientations of the joint surfaces. In each sagittal view (grooves and ridge), the articular surface of the patella was divided into quarters, resulting in three ROIs per slice.

The cartilage of the femoropatellar joint was evaluated on the trochlear ridges, in the trochlear groove, and on the facies articularis of the patella (Figure 1, Figure 2, Figure 3 and Figure 4).

Articular cartilage was measured in the sagittal and transverse MR images. In total, 18 regions of interest (ROI) were determined; three each on the lateral and medial trochlear ridge of the femur, and three in the trochlear groove of the femur. On the facies articularis of the patella, three ROIs were determined each in the lateral and medial groove of the patella and on the central ridge of the patella (Figure 4). In each ROI, the total cartilage thickness measurements were obtained three times and recorded in an excel data sheet.

#### 2.3.2. Femorotibial Joint

**Figure 5:**
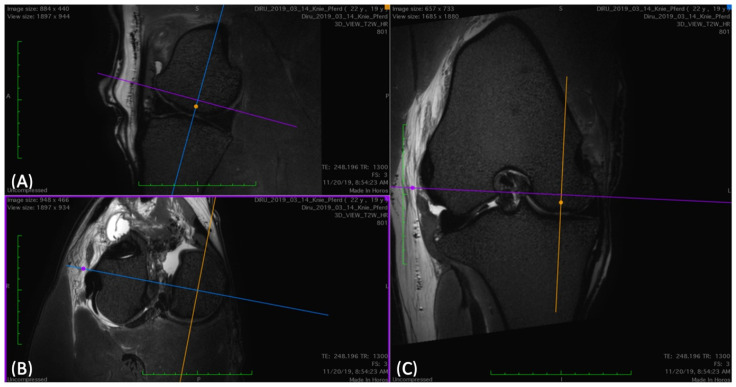
(**A**) Sagittal, (**B**) transverse, and (**C**) dorsal view of 3D T2-weighted view images of a stifle, where the plane for the regions of interest (ROIs) is determined for the medial femorotibial joint. To have a sagittal view directly through the middle of each condyle, the dorsal plane (blue) was aligned in parallel to the caudal horn of the meniscus and the transverse (purple) parallel to the joint space. In the dorsal (**C**) and transverse (**B**) views, the sagittal plane (yellow) was placed through the center of the condyle. This was repeated for lateral measurements.

**Figure 6:**
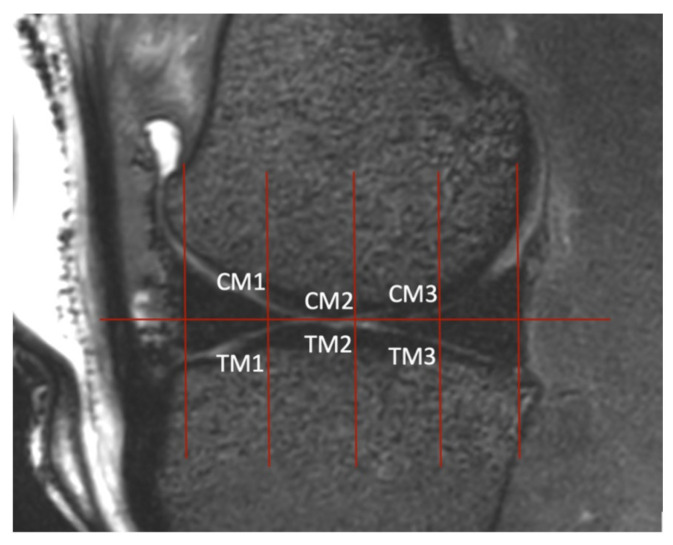
Sagittal view of the medial femorotibial joint in a 3D T2-weighted VIEW image where the regions of interest (ROIs) were determined. The sagittal plane was set as described in Figure 5. In this view, two lines were drawn. The first one passed vertically through the largest extent of the cranial aspect of the meniscus and the second passed through the caudal aspect. The distance between these two lines was quartered by drawing three lines that were parallel to the first two. Where these lines intercepted the cartilage, the thickness was measured (ROIs CM1, CM2, CM3, TM1, TM2, TM3). This was carried out for the lateral femorotibial joint as well.

The femoral condyles and tibial cartilage were assessed in the sagittal and dorsal views (Figure 5 and Figure 6). A cranial, center, and caudal ROI was determined for both the tibial surface and the condyles. This resulted in six ROIs each for the lateral and medial femorotibial joints. As in the femoropatellar joint, the cartilage thickness was also measured three times at each ROI and recorded in the excel data sheet.

### 2.4. Macroscopic Measurements

For the macroscopic measurements, the limbs were carefully dissected by one of the authors (A.S.): using a large band saw, the stifle joint was isolated from the rest of the limb by cutting the femur just proximal to the trochlea and the tibia distal to the tibial plateau. To visualize the cartilage thickness, thin slices were created using an anatomic band saw. First the femoropatellar and the femorotibial joints were separated from each other, just at the transition from the femoral condyle to the trochlea. Then, cuts were made sagittally through the trochlear ridges and the groove. The same was carried out for the patella. Each condyle and corresponding joint surface of the tibia was sliced through the center. These slices were then scanned using a flatbed color image scanner (Epson Perfection V700 Photo scanner, Amsterdam, The Netherlands) at a resolution of 800 dpi (dots per inch). In each scan, a precise ruler was included as a reference. Articular cartilage thickness was measured at the determined ROIs (see image analysis) and recorded with a dedicated open source image processing software (ImageJ 2.0.0, National Institute of Health, Bethesda, MD, USA).

**Figure 7:**
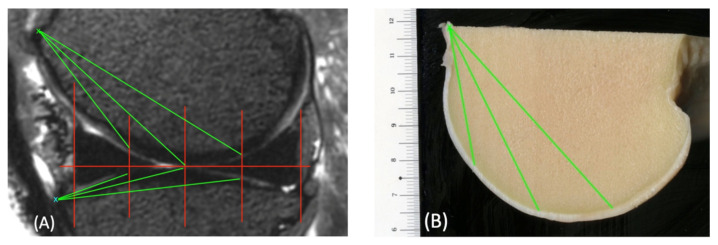
Sagittal view of the medial femorotibial joint in a 3D T2-weighted VIEW sequence (**A**) and the condyle in a macroscopic scan (**B**), with marked regions of interest (ROIs, red lines). The base of the condyle (green x) is marked. From this point, the distances to each ROI were measured (green lines). This distance could then be used to find the corresponding ROIs in the macroscopic scans. The same was carried out for the tibia using the tuberositas tibiae (blue x) as the reference point.

The base of the femur trochlea was used as a reference point from which the distance to the ROIs was measured in the MRI images (Figure 7). This was then used in the macroscopic images to find the corresponding ROI.

### 2.5. Statistical Analysis

**Figure 8:**
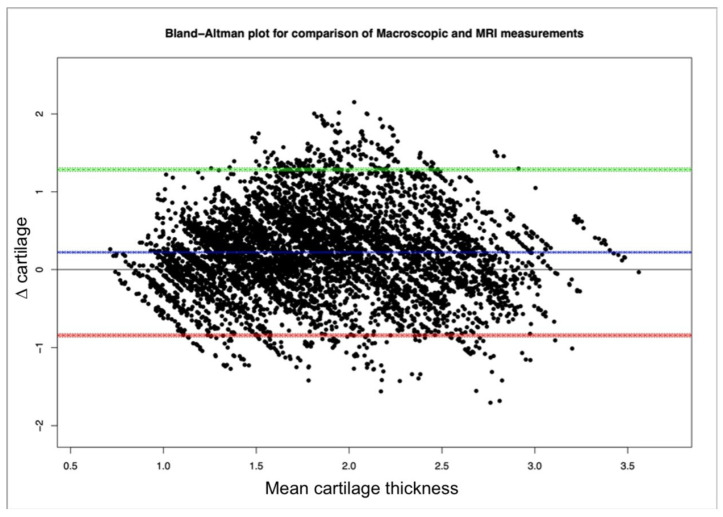
Bland–Altman plot comparing macroscopic and MRI measurements. The plot shows ∆ cartilage (mean difference macroscopic to MRI measurements) plotted against the mean cartilage thicknesses. The horizontal blue line is the mean ∆ cartilage and the green and red lines are the upper and lower limits of agreement. The mean ∆ cartilage is 0.221 mm, which is significant (*p* < 0.0001).

The database was established in Microsoft Excel. The distribution of data for continuous variables was assessed for normality by use of the Kolmogorov–Smirnoff test. Results were reported as the mean ± standard deviation for variables with a parametric distribution (and median (range) for variables with nonparametric distributions). The mean differences between the measurements of the macro slices and those of the MRI sequences were calculated. This mean difference was referred to as ∆ cartilage. ∆ cartilage was analyzed using a Bland–Altman plot, plotting the ∆ cartilage against the mean cartilage thickness (Figure 8). A multivariable regression model with random effects was performed to determine which factors affected the differences in ∆ cartilage. Random effects was used as there were repeated measurements for each ROI. All statistical analyses were performed with commercially available statistical software programs (R Core Team (2016); R: A language and environment for statistical computing; R Foundation for Statistical Computing, Vienna, Austria; URL: https://www.R-project.org (accessed on 23 September 2021); using the MASS, car and lme4 packages and SPSS Inc., Chicago IL, USA) and a *p*-value < 0.05 was considered statistically significant.

## 3. Results

Seven stifles (three left and four right limbs) of seven warmblooded horse cadavers (four mares and two geldings) with a mean age of 16.7 ± 5.06 years (9–22 years) were obtained. Of the seven stifles included in the study, six could be evaluated completely macroscopically and using the MRI sequences. In one stifle, the proximal aspect of the medial trochlea was cut off so that the proximal measurement (ROI FM1) was not possible.

The mean overall macroscopic cartilage thickness (1.944 ± 0.578 mm) was significantly greater than the mean overall cartilage thickness measured on MRI (1.718 ± 0.581 mm) (*p* < 0.0001) (Figure 8). The mean difference (∆ cartilage) for all measurements was 0.221 ± 0.543 mm.

### 3.1. Two-Dimensional Analysis (T2W TSE)

An overview of the cartilage thickness measurements obtained for each ROI in the 2D T2W TSE MR sequence and the corresponding macroscopic ROIs is shown in Table 2.

The overall ∆ cartilage between the macroscopic cartilage thickness (1.944 ± 0.578 mm) and the 2D MR thickness (1.477 ± 0.53 mm) was 0.465 ± 0.569 mm. This was significantly greater than in all other sequences evaluated (*p* < 0.001). Within the T2W TSE sequence the difference between sagittal, dorsal, and transverse measurements was not significantly different (*p* = 0.75 − 1). The transverse measurements apply to the femoropatellar joint and the dorsal measurements to the femorotibial joint.

### 3.2. Three-Dimensional Analysis (3D VIEW T2W HR, 3D PDW SPAIR, T2 3D mFFE, and T1W VISTA SPAIR)

An overview of the cartilage thickness measurements obtained for each ROI in the 3D MRI sequences and the corresponding macroscopic ROIs is shown in Table 2.

When analyzing the 3D sequences, the 3D VIEW T2W HR (0.137 ± 0.525 mm) and T1W VISTA SPAIR (0.111 ± 0.466 mm) sequences had the smallest ∆ cartilage compared to 3D PDW SPAIR (0.212 ± 0.483 mm, *p* < 0.001) and T2 3D mFFE (0.181 ± 0.586 mm, *p* < 0.001). Both 3D VIEW T2W HR and T1W VISTA SPAIR were not statistically significantly different in ∆ cartilage (*p* = 0.222). Likewise, 3D PDW SPAIR and T2 3D mFFE were also not significantly different in terms of ∆ cartilage (*p* = 0.72).

Within the 3D PDW SPAIR sequence, there was no statistically significant difference between the measurements in the sagittal, transverse, and dorsal views (*p* = 0.6–1). In the 3D VIEW T2W HR and T1W VISTA SPAIR sequences, there was also no significant difference between ∆ cartilage in sagittal and transverse views (*p* = 1 for both). However, ∆ cartilage for both was significantly smaller than in the dorsal (*p* < 0.05). In the T2 3D mFFE sequence, the ∆ cartilage on dorsal measurements was significantly thinner than on both the sagittal and transverse planes (*p* < 0.001). Subsequently, the ∆ cartilage of measurements on the sagittal plane were thinner than the ∆ cartilage of measurements obtained on the transverse plane (*p* < 0.001).

In the multivariable regression, model joints (femorotibial or femoropatellar) did not significantly affect ∆ cartilage for any sequence (*p* = 0.6).

In the femur, ∆ cartilage for all MRI sequences (0.0959 ± 0.509 mm) was significantly thinner than ∆ cartilage of the patella (0.322 ± 0.554 mm) (*p* < 0.001) and of the tibia (0.377 ± 0.535 mm) (*p* < 0.001). The patella had a slightly smaller ∆ cartilage than the tibia (*p* = 0.004). In the femorotibial joints, the laterally (0.161 ± 0.478 mm) situated ∆ cartilages were significantly smaller than the medial ones (0.336 ± 0.518 mm) (*p* = 0.016). In the femoropatellar joint, the ∆ cartilages located axially in the joint (0.113 ± 0.56 mm) were significantly smaller than those laterally (0.318 ± 0.533 mm) and medially (0.179 ± 0.585 mm) (*p* < 0.001 and *p* = 0.009, respectively), while medially situated mean differences were significantly smaller than the lateral ones (*p* < 0.001).

In the femoropatellar joint, ∆ cartilage of the proximal ROIs (0.1668 ± 0.594 mm) was thinner than the middle ROIs (0.24 ± 0.517 mm) (*p* < 0.001). The distal ROI (0.204 ± 0.582 mm) was not significantly different to the others.

In the femorotibial joint, the center measurements had a ∆ cartilage of 0.36 ± 0.444 mm. This was significantly thicker than the cranial (0.077 ± 0.562 mm, *p* < 0.001) and caudal (0.307 ± 0.456 mm *p* = 0.019) ∆ cartilage. The cranial ∆ cartilage was significantly thinner than the caudal (*p* < 0.001).

## 4. Discussion

Despite being established in humans, as a noninvasive diagnostic technique for evaluating articular cartilage [1,2], the MR imaging of stifle cartilage has not been established in the horse. With advancing technology and stifle MRI becoming more readily available in equine clinics, this needs to be established, since it offers the noninvasive evaluation of not only articular cartilage but also other articular and periarticular structures which cannot be evaluated via other imaging modalities [2,3,4]. The authors of the investigation hypothesized that the 3D sequences would be at least equal to the 2D sequence in depicting cartilage. This assumption was the case, since all 3D sequences were significantly more accurate than the 2D sequence used in this study.

One of the most commonly used sequences for depicting cartilage is 2D T2w TSE [2,3,4,5,6]. Although the sequence has been shown to have good in plane accuracy in depicting cartilage, there are some limitations: the slice thickness is thicker compared to 3D sequences which can lead to partial volume-averaging artifacts and difficult multiplanar reconstruction [2,3,4,5]. In the present study, cartilage thickness was measured in different planes for the same ROI. For 3D sequences, simultaneous evaluation in the multiplanar reconstruction of all planes was direct and possible within the same image sequence. For 2D sequences, each plane has to be acquired and reviewed individually. Therefore, it is not possible to reliably measure in the multiplanar reconstruction, due to substantial image quality loss in all but the plane the sequence was acquired in. Measurements had to be performed in each separate sequence, which may reduce the accuracy of where each measurement was made. Together with the increased slice thickness, this may account for the differences seen between 2D and 3D measurements.

Of the 3D sequences, 3D VIEW T2W HR and T1W VISTA SPAIR had the smallest differences to macroscopic measurements. The 3D VIEW T2W HR is a high resolution T2-weighted 3D fast spin-echo sequence. T1W VISTA SPAIR is a T1-weighted TSE sequence with fat suppression. The 3D PDW SPAIR and T2 3D mFFE were less accurate.

All 3D sequences used in the present study have qualities that have been described in cartilage imaging. T2w and PDw FSE and TSE sequences have shown excellent contrast between fluid and cartilage and are most commonly used in cartilage depiction [2,3,4,5]. T1-weighted sequences are described to have better subchondral bone to cartilage contrast [3]. This is enhanced by using fat suppression, in this case, spectral attenuated inversion recovery (SPAIR) [2]. Apart from the FSE and TSE sequences, GRE sequences have also been described in cartilage imaging [3,7]. GRE is an advanced sequence with a high signal-to-noise ratio and good cartilage surface depiction. Limitations are the higher susceptibility to motion artifacts and low signal changes depending on cartilage health [3,7]. T2 3D mFFE is the GRE sequence used in this study, and it was less accurate than 3D VIEW T2W HR and T1W VISTA SPAIR.

On average, the macroscopic measurements were significantly greater than the MRI measurements. The average difference was 0.221 mm. This has previously been described in horses and humans [8,9,10]. A reason for this was speculated to be that the calcified portion of cartilage may be considered subchondral bone in MRI and thus not included in the cartilage thickness measurement [9]. However, a more recent study found that macroscopic measurements including the calcified layer were more accurate compared to MRI measurements than if only the hyaline cartilage thickness was considered [11]. In a different human study, it has been found that generally thinner cartilage (<2.5 mm) is overestimated in MRI and thicker cartilage (>3.5 mm) is underestimated. In between 2.5 mm and 3.5 mm thickness, the accuracy was the highest [12]. These different accuracies have to do with not only cartilage thickness but also joint curvature, spatial resolution, and the acquisition parameters [3]. In a study on equine metacarpalphalangeal joints, it was speculated that magnetic susceptibility artifacts may cause cartilage to be especially underestimated where articular surfaces are directly opposing [10].

When evaluating the accuracy, according to the bone, the femur had the smallest ∆ cartilage and the tibia the largest with the patella being in between. It has been described that it is harder to distinguish between opposing cartilage surfaces or between cartilage and meniscus, which may explain the lower accuracy for the tibia [13,14]. In the present study, this lower accuracy was seen in the center and caudal measurements of the femorotibial joint. In previous human studies, the lateral compartment has been described to have lower diagnostic accuracy as opposed to the medial and femoropatellar joint compartments. This was thought to be due to the convex surface of the lateral tibial plateau [6,15]. Our findings show a higher accuracy for the lateral compartment compared to the medial. This may have to do with the anatomy in the equine stifle being slightly different than the human knee. It is also important to consider that the MRI acquisition is a non-weight-bearing position, making joint space characteristics less accurate. In the femoropatellar articular cartilage, measurements in the groove were most accurate. This may be due to the surface of the groove being less curved than those of the trochleae [3]. Proximal measurements had less deviation from macroscopic cartilage thickness. Most deviation was found middle to distally in the joint; this may have to do with proximal articular cartilage being slightly thicker than distal articular cartilage [3,16].

Depending on the sequence, individual planes were more accurate or advantageous than others. Within the 3D PDW SPAIR sequence, there was no statistically significant difference between the planes. In the 3D VIEW T2W HR and T1W VISTA SPAIR sequences, there was also no significant difference between ∆ cartilage in sagittal and transverse views, with the dorsal measurements being less accurate. In the T2 3D mFFE, dorsal measurements were the most accurate, followed by the sagittal and then the transverse plane. As previously stated, the transverse measurements apply to the femoropatellar joint and the dorsal to the femorotibial joint. To the authors’ knowledge, no other studies describe a comparison between these planes.

This study has some limitations. In spite of using measurements to determine the corresponding ROIs, it is still possible that there are small deviations of where the cartilage was measured macroscopically and in MRI. Inaccuracies are more likely in the 2D sequence, since the direct measurements of the same ROI in different planes of a multiplanar reconstruction is not possible. A consensus between two observers was reached when measurements were obtained and no interobserver agreement could be determined. Due to the arthroscopic exploration of the stifle joints prior to imaging, we were certain that the cartilage was healthy. Air artifacts were not a problem for the evaluation of the cartilage. The scanner used for the macroscopic slices has been used in a previous study and has proven to have high image quality sufficient for the exact measurements of the cartilage [17].

## 5. Conclusions

In spite of the unavoidable limitations described in the discussion, this study showed that 3D sequences had a higher accuracy in depicting cartilage than 2D sequences, with 3D VIEW T2W HR and T1W VISTA SPAIR obtaining the closest measurements compared to gross pathology. It is likely that this higher accuracy in 3D images is largely due to thinner slices in 3D scans and the possibility of precisely measuring the same ROI in different planes through multiplanar reconstruction. Generally, it is important to consider multiple planes when evaluating MRI images, since no plane was consistently more accurate than the others. As described in other studies, the least accurate measurements were obtained where cartilage or meniscus had opposing surfaces, making it difficult to differentiate the exact margins [10,13,14]. Three-dimensional sequences are more than sufficient to replace traditional two-dimensional imaging. This would be especially useful while scanning horses since imaging time is significantly shorter [3,4,15]. Stifle MRIs are becoming more available, and knowing the accuracy of different sequences will improve the evaluation of equine cartilage and the early detection of cartilage pathologies.

## Figures and Tables

**Table 1:** MRI sequence parameters used in the study. Abbreviations: FOV: field of view; TR: time to repeat; TE: time to echo.

	T2W_TSE_Sag	T2W_TSE_Cor	T2W_TSE_Tra	3D_View_T2W_HR	3D_PDW_SPAIR	T2_3D_mFFE	T1W_VISTA_SPAIR
**Plane**	sagittal	dorsal	transverse	sagittal	sagittal	sagittal	sagittal
**Reconstructed voxel size (mm)**	0.391 × 0.391	0.228 × 0.228	0.196 × 0.196	0.458 × 0.458 × 0.55	0.174 × 0.174 × 0.7	0.391 × 0.391 × 1	0.289 × 0.289 × 0.35
**Slice thickness (mm)**	3	3	3	0.55	0.70	2	0.7
**FOV (mm)**	250 × 250 × 181	230 × 202 × 188	220 × 220 × 221	220 × 202 × 300	200 × 250 × 300	200 × 200 × 300	250 × 199 × 300
**Matrix**	416 × 375	384 × 313	340 × 334	276 × 253	288 × 357	332 × 335	356 × 239
**TR**	4557	4674	3737	1300	1200	31	350
**Frequency offset**	no	no	no	no	default	no	220
**TE**	80	80	80	257	194	9.2	19
**Averages**	1	1	1	1	1	1	1
**Interslice gap (mm)**	3	3	3	−0.3	0	−1	−0.35
**Flip angle**	90	90	90	90	90	90	90

MRI sequence parameters used in the study. Abbreviations: FOV: field of view; TR: time to repeat TE: time to echo.

**Table 2:** Mean cartilage thickness measurements of each region of interest (ROI) in mm for each MRI sequence and for the macroscopic evaluation of equine cadaver stifle joints. Abbreviations: FM = femur medial; FC = femur central; FL = femur lateral; PM = patella medial; PC = patella central; PL = patella lateral; CM = condyle medial; CL = condyle lateral; TM = tibia medial; TL = tibia lateral; 1 = proximal; 2 = mid; 3 = distal; 4 = cranial; 5 = central; 6 = caudal.

ROI	T2W TSE	3D VIEW T2W	3D PDW SPAIR	T2 3D mFFE	T1W VISTA SPAIR	Macroscopic Analysis
**FM1**	1.149	1.236	1.305	1.278	1.291	0.942
**FM2**	1.074	1.458	1.409	1.457	1.429	1.295
**FM3**	1.444	1.881	1.692	1.456	1.717	1.767
**FC1**	1.930	2.068	2.171	2.258	2.115	2.115
**FC2**	1.523	1.981	1.865	2.115	1.876	1.950
**FC3**	1.936	2.456	2.330	1.687	2.322	1.833
**FL1**	2.168	1.873	2.111	1.418	2.080	2.287
**FL2**	1.874	1.779	1.832	1.663	1.781	2.232
**FL3**	1.793	2.051	2.292	1.742	2.075	2.351
**PM1**	1.174	1.757	1.537	1.525	1.990	1.963
**PM2**	1.580	2.548	2.200	1.979	2.535	2.449
**PM3**	1.816	2.139	2.382	2.572	2.650	2.910
**PC1**	1.540	2.059	1.788	1.789	2.117	2.127
**PC2**	1.650	2.411	2.186	1.953	2.541	2.488
**PC3**	1.722	2.569	2.321	2.215	2.412	2.547
**PL1**	1.793	2.595	2.276	1.956	2.447	2.444
**PL2**	2.009	2.639	2.444	2.289	2.694	2.780
**PL3**	1.918	2.219	2.171	2.201	2.492	2.349
**CM4**	1.253	1.559	1.435	1.714	1.546	1.626
**CM5**	0.948	1.157	1.215	1.637	1.322	1.472
**CM6**	1.278	1.428	1.428	1.492	1.430	1.638
**CL4**	1.334	1.533	1.234	1.453	1.214	1.219
**CL5**	1.096	1.217	1.062	1.114	1.069	1.403
**CL6**	1.177	1.377	1.149	1.246	1.148	1.227
**TM4**	1.279	1.491	1.471	2.170	1.866	2.114
**TM5**	0.951	1.209	1.231	1.856	1.478	1.914
**TM6**	1.137	1.297	1.218	1.472	1.246	1.706
**TL4**	1.374	1.551	1.512	2.095	1.614	1.488
**TL5**	1.204	1.318	1.390	1.674	1.276	1.735
**TL6**	1.176	1.206	1.155	1.257	1.073	1.751

## Data Availability

Data are contained within the article.
